# Conservative Management of Low Back Pain and Scoliosis in a Patient With Rheumatoid Arthritis: Eight Years Follow-Up

**DOI:** 10.7759/cureus.36036

**Published:** 2023-03-12

**Authors:** Eric Chun-Pu Chu, Hay Yeung Cheng, Kevin Huang, Kristy Yao, Jason Zhao

**Affiliations:** 1 New York Medical Group (NYMG) Chiropractic Department, EC Healthcare, Hong Kong, HKG; 2 New York Medical Group (NYMG) Chiropractic Department, EC Healthcare, Yuen Long, HKG; 3 New York Medical Group (NYMG) Chiropractic Department, EC Healthcare, Taikoo, HKG

**Keywords:** chiropractic management, chiropractic therapy, inflammatory disease, scoliosis, rheumatoid arthritis

## Abstract

Scoliosis in patients with rheumatoid arthritis (RA) can cause significant pain and disability. RA has been extensively studied in relation to the cervical spine, yet the pathology of the thoracic and lumbar spine in RA patients has been largely overlooked. A 66-year-old woman, with longstanding RA and severe scoliosis, presented to the chiropractic clinic with a five-month history of exacerbated low back pain radiating to the right lower limb. The patient was treated with a combination of full-spine mechanical spinal distraction, spinal manipulative therapy, mechanical distraction of the cervical spine, and soft tissue treatment (scraping therapy). Thereafter, the patient recovered from the pain and radiculopathy and showed improvements in the radiological parameters, walking gait, and postural balance. Radiography was performed at the 12-month, four-year, and eight-year follow-up appointments and revealed improvements in symptoms, posture, and scoliosis. Although the treatment for RA-related scoliosis is similar to that for other types of scoliosis, due to the nature of RA, treatment should be tailored to individual patients. This case report highlights the importance of considering chiropractic therapy for the management of lumbar scoliosis in patients with RA, as a comprehensive treatment plan resulted in improved spinal balance, mobility, gait, posture, and quality of life.

## Introduction

Rheumatoid Arthritis (RA) is an inflammatory autoimmune disease characterized by the widespread swelling joints of multiple joints, including the atlantoaxial ligament of the cervical spine. The craniocervical junction is composed solely of ligaments and synovial joints, making it susceptible to the inflammatory process of RA [1]. Inflammatory processes in RA can cause asymmetric arthritis of the facet joints, costovertebral joints, and vertebral bodies, resulting in spinal deformities [2]. RA has been extensively studied in relation to the cervical spine, yet the pathology of the thoracic and lumbar spine in RA patients has been largely overlooked. It is estimated that between 30-50% of RA patients experience back pain, leading to greater levels of disability and depression compared to those who do not experience back pain [3]. Recent studies have revealed that individuals with RA suffer from a higher rate of disc narrowing, subluxation, osteoporosis, and apophyseal destruction of the lumbar spine in comparison to individuals who do not have RA. These conditions can lead to long-term complications and severe pain in the lower back region. Early diagnosis, prompt medical treatment, and lifestyle modifications are essential to ensure the best possible outcomes [4]. The treatment for RA-related scoliosis is similar to that for other types of scoliosis and includes bracing, physical therapy, and surgery. However, due to the nature of RA, treatment should be tailored to individual patients.

Chiropractors are healthcare providers who use manual therapy to treat neuromusculoskeletal conditions [5]. Many case reports have suggested that chiropractic therapy may help manage spinal conditions related to the symptoms of RA [6, 7]. Chiropractic care includes hands-on spinal manipulation and mobilization for alleviating pain and improving range of motion, as well as the prescription of rehabilitative exercises. Most existing literature focuses on cervical spine involvement in RA, but this patient had severe thoracolumbar scoliosis. The effects of RA on the lower spine and management of resulting scoliosis deserve more study. This highlights the importance of our case report, which provides an eight-year follow-up of the clinical presentation and chiropractic care of thoracolumbar spine pathology in a patient with RA, contributing to the current knowledge of patient management.

## Case presentation

**Figure 1:**
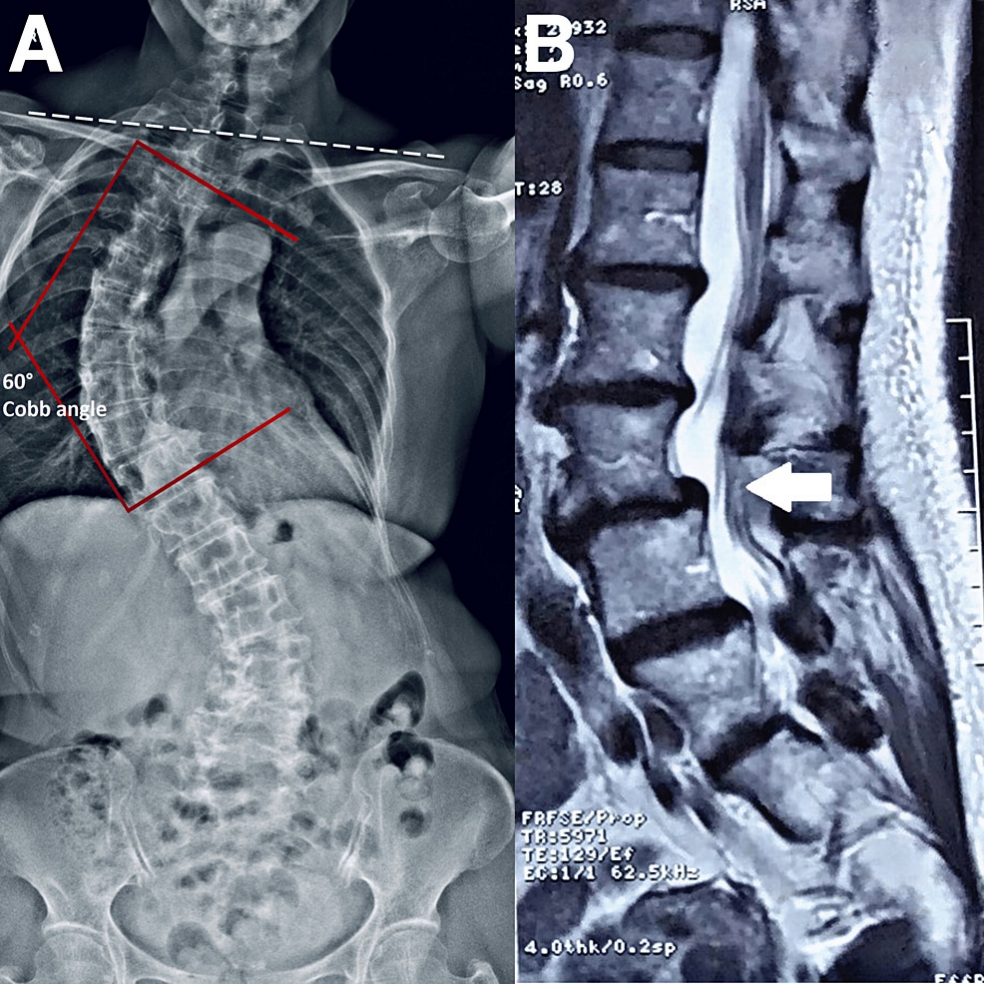
Full-spine radiograph and lumbar magnetic resonance image A) Full-spine radiograph revealing degenerative changes in the thoracic and lumbar regions, shoulder and pelvic imbalance (dash white line), and abnormal thoracic and lumbar scoliotic curves. The Cobb angle was measured at 60 ° (red lines) B) At L4-5 (white arrows), there are spondylolisthesis, posterior bulging disc, and marked bilateral facet joint degeneration, in the right facet joint and ligamentum flavum hypertrophy causing mild indentation of the thecal sac. Diffuse bulging disc and osteophytes at L2-3 causing mild indentation of the thecal sac, mild right lateral recess and mild bilateral foraminal stenosis. No nerve root compression is seen.

A 66-year-old female office administrator presented with a five-month history of exacerbated low back pain, and longstanding RA and scoliosis. She experienced sharp, radiating pain extending to her right sacroiliac joint and down her right leg. The pain pulled on her leg and limited her mobility, often requiring her to use a cane. Even with a cane, she could only walk for approximately 20 min before being limited by severe pain. These symptoms negatively impacted her sleep quality and social life. She rated her average pain severity as 6 out of 10 and stated that the symptoms were stress-related. Her World Health Organization Quality of Life (WHOQOL) assessment score was 70%. Radiography (Figure 1A) and MRI (Figure 1B) showed degenerative disc disease, severe scoliosis, and spondylolisthesis at L3/4. She was administered pain medication and acupuncture with minimal improvement. Her orthopedist recommended surgical intervention. She sought a second opinion and non-surgical options to help manage the pain and complications from worsening scoliosis.

Her medical history revealed that she had osteoporosis, RA, and insomnia. The moderately severe RA, which required continued treatment with leflunomide, methotrexate, and prednisone, was discovered 20 years earlier. Her scoliosis over the course of 10 years progressively worsened by 10 degrees at the same time as her RA diagnosis. Additionally, she underwent a thyroidectomy for hyperthyroidism six years prior, and levothyroxine was prescribed for post-thyroidectomy care. She denied having saddle anesthetic or losing control of her bladder, despite having a family history of scoliosis and RA. She had been swimming once a week to keep her musculoskeletal system in good shape. The patient provided written authorization for the use of her photographs, and the study received approval from the appropriate ethics committee.

**Figure 2:**
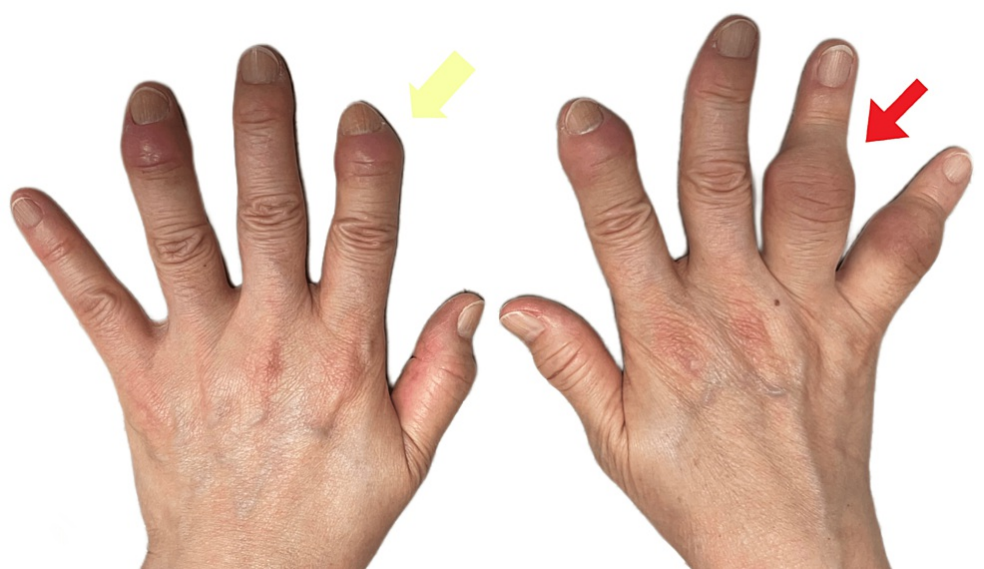
Photo of patient's hands Her hands show Heberden (yellow arrow) and Bouchard nodes (red arrow) on the fingers.

On physical examination, she weighed 65 kg and measured 160 cm in height, with an abnormal kyphotic posture that caused an imbalance, shoulder heaviness, and constant tightness across her upper back. Heberden and Bouchard's nodes were observed on her fingers (Figure 2). She had a limited range of motion in all planes in her lumbar spine due to pain and stiffness. Palpable muscle spasms and tenderness were present over the lumbar spine and right sacroiliac joint. Neurologically, the sensation was intact and reflexes were equal bilaterally. She had a negative straight leg raise test. The results of her Adam’s test showed abnormal kyphotic curvature with an elevated right shoulder, uneven waistline, and rib hump, indicating asymmetrical trunk rotation. Her lower extremities showed no edema and normal muscle tone/strength, although her right ankle jerk reflex was diminished. She had decreased sensation on her right distal leg and ankle, and tenderness over her right greater trochanter and lateral knee. Standing posture and limb position measurements indicated a shortened right leg and pelvic tilt. On palpation, spinal intersegmental dysfunction was identified from T3-5, T7-T8, and L1-L2. Bilateral hypertonicity was observed at the quadratus lumborum, rectus femoris, and erector spinae. Functionally, she ambulated with a limp, favoring her right leg, and used a cane for prolonged walking and community mobility. She mobilized in and out of a chair slowly and with caution due to her limited spinal range of motion, which impacted her daily tasks. Based on her clinical history and radiological results, she is diagnosed with degenerative scoliosis complicated by spondylolisthesis and rheumatoid arthritis.

**Figure 3:**
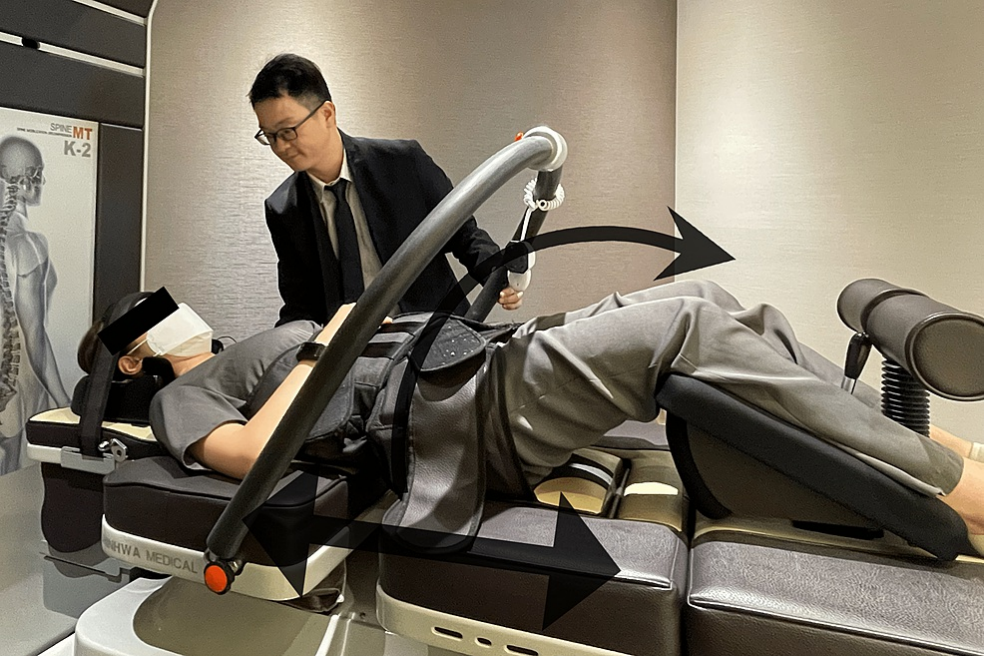
Mechanical spinal distraction demonstration on scoliosis The patient is positioned in supine on the distraction device (Spine MT, Shinhwa Medical, Korea) with the head, thoracic, and pelvis stabilized via straps. The knees are in flexed position. A distraction and rotation force (20% of the body weight) is applied for 15 minutes.

Chiropractic intervention was used to address the patient's scoliosis, as well as any associated shoulder and pelvic imbalances. The treatment involved a combination of techniques, including full-spine mechanical spinal distraction, spinal manipulative therapy, the mechanical spinal distraction of the cervical spine, and soft tissue treatment (scraping therapy). During the sessions, the chiropractor applied manipulation at the apex of the scoliotic curve and to the restricted vertebral segments to correct the spinal curvature. Mechanical spinal distraction is used to restore the restore scoliotic curves (Figure 3) and scraping therapy on the hypertonic soft tissues to stabilize the spine. The patient received three treatment sessions per week for the first month and was given lifestyle coaching to help them maintain proper posture and spinal alignment. The range of motion in her back improved after four weeks of treatment, and specific tight muscle stretching was performed in each session. The treatments were continued twice per week for an additional five months, resulting in improved spinal mobility, gait, and trunk posture. Continued chiropractic care and custom foot orthotics were administered to improve abnormal motion within the pelvis and correct leg length discrepancy.

**Figure 4:**
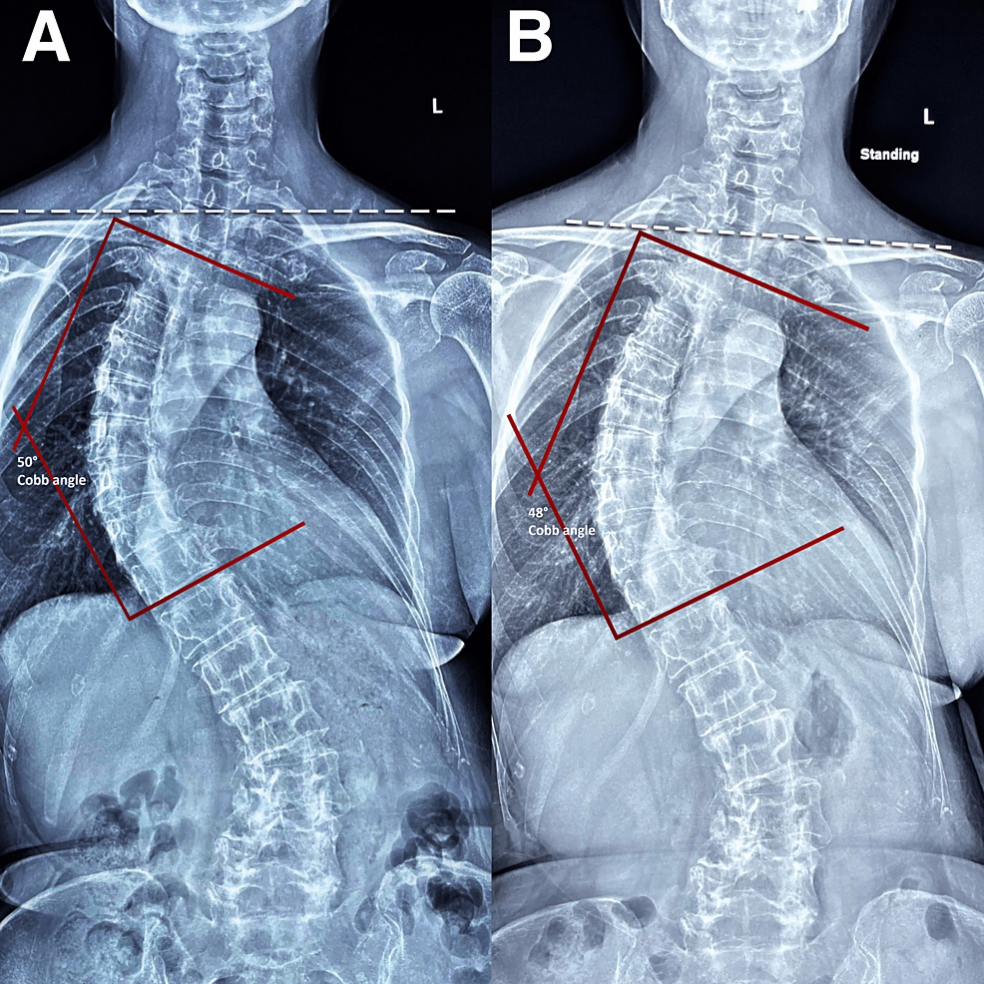
Full-spine radiographs at the 12th month and eighth year re-evaluations A) At the 12th month, radiographs showed improved spinal curvature and posture, as well as halted scoliosis progression. Shoulder is more balanced, and the Cobb angle was reduced from 60 ° to 50° (red lines) B) At the 8th year, the thoraco-lumbar scoliosis and shoulder (dash white line) and pelvic imbalances showed improvement.  The Cobb angle was stable at 48 ° (red lines)

At 12 months, radiographs revealed normalized spinal curvature and posture and halted scoliosis progression (Figure 4A). The patient reported symptom resolution, and her WHOQOL score improved to 98% (from 70%). The patient's treatment schedule was reduced to monthly maintenance appointments to preserve the spinal alignments [8]. Follow-up radiographs at four and eight years confirmed a stable spinal curvature, and the patient was symptom-free (Figure 4B). Her WHOQOL score remained at 98% and no adverse events were observed.

## Discussion

In total, 32.0% of patients with RA have lumbar scoliosis [9]. The severity of scoliosis in patients with RA depends on disease duration and severity, the pattern of joint involvement, genetics, osteoporosis, weight, fatigue, and muscle weakness. Longer duration and higher disease activity or severity of RA increase the risk of joint damage, leading to scoliosis [10]. If the joints and ligaments connecting the spine to the pelvis are severely affected, the risk of spinal instability and deformity increases [11]. Family history may play a role in the developmental risk of scoliosis from RA [12]. Chronic inflammation in RA can lead to osteoporosis, which causes bone fragility [13]. Chronic pain and fatigue due to RA can lead to decreased mobility and muscle weakness, contributing to scoliosis [14]. The presence of RA in patients with scoliosis can exacerbate spinal curve progression, leading to increased pain, disability, and decreased quality of life.

Additionally, immunosuppressive medications commonly used in the management of RA, such as methotrexate and prednisone, can weaken vertebral bodies and contribute to the progression of scoliosis [15]. As our patient had a family history of scoliosis and RA, it was crucial for her healthcare practitioners to closely monitor spinal health in osteoporosis and chronic pain and fatigue symptoms to prevent or manage spinal curve progression and improve overall health outcomes.

Chiropractic treatment has been shown to be effective in managing RA-related symptoms. Several studies have reported significant improvements in pain, joint mobility, and overall health-related quality of life in patients with RA undergoing chiropractic care [6, 7]. Evidence from a systematic review of alternative medicine for RA indicates that many therapies may be useful for treating rheumatic diseases [16]. In addition, physical therapy modalities, such as exercise, can improve muscle strength and flexibility, reducing the risk of joint damage and improving overall joint function in older adults with RA [17]. These findings suggest that chiropractic care can be complementary to traditional medical management of patients with RA, providing significant improvements in low back pain and overall health outcomes.

Chiropractors also manage scoliosis-related symptoms including spinal subluxation and low back pain. Several studies have reported significant improvements in pain, posture, and overall spinal alignment in patients with scoliosis undergoing chiropractic care [18, 19]. Spinal manipulative therapy has been shown to reduce pain and improve spinal alignment in patients with scoliosis [20]. In addition, corrective and therapeutic exercise can improve function and reduce symptoms in patients with scoliosis, thereby reducing the risk of spinal injury and improving overall spinal function [21]. Adult patients with scoliosis with mild-to-moderate (17-26°) curve magnitudes can have mild reductions in unusually high thoracic curves [18], and adolescent idiopathic scoliosis can have more reduction through chiropractic care [19]. These findings suggest that chiropractic care can be an effective alternative to traditional medical management for scoliosis in patients with RA, providing significant improvements in pain and overall spinal health.

## Conclusions

This case report highlights the effectiveness of a comprehensive treatment strategy during an eight-year follow-up, which included chiropractic care, the prescription of orthotics, and lifestyle changes, in the successful management of lumbar scoliosis in a patient with RA. More research is required to demonstrate the efficacy of this method in a wider patient group due to the limitations of a single case report.
